# Detection of Intracellular Bacterial Communities in Human Urinary Tract Infection

**DOI:** 10.1371/journal.pmed.0040329

**Published:** 2007-12-18

**Authors:** David A Rosen, Thomas M Hooton, Walter E Stamm, Peter A Humphrey, Scott J Hultgren

**Affiliations:** 1 Department of Molecular Microbiology and Microbial Pathogenesis, Washington University School of Medicine, St. Louis, Missouri, United States of America; 2 Department of Medicine, University of Miami Miller School of Medicine, Miami, Florida, United States of America; 3 Department of Medicine, University of Washington School of Medicine, Seattle, Washington, United States of America; 4 Department of Pathology and Immunology, Washington University School of Medicine, St. Louis, Missouri, United States of America; Brown University School of Medicine, United States of America

## Abstract

**Background:**

Urinary tract infections (UTIs) are one of the most common bacterial infections and are predominantly caused by uropathogenic Escherichia coli (UPEC). While UTIs are typically considered extracellular infections, it has been recently demonstrated that UPEC bind to, invade, and replicate within the murine bladder urothelium to form intracellular bacterial communities (IBCs). These IBCs dissociate and bacteria flux out of bladder facet cells, some with filamentous morphology, and ultimately establish quiescent intracellular reservoirs that can seed recurrent infection. This IBC pathogenic cycle has not yet been investigated in humans. In this study we sought to determine whether evidence of an IBC pathway could be found in urine specimens from women with acute UTI.

**Methods and Findings:**

We collected midstream, clean-catch urine specimens from 80 young healthy women with acute uncomplicated cystitis and 20 asymptomatic women with a history of UTI. Investigators were blinded to culture results and clinical history. Samples were analyzed by light microscopy, immunofluorescence, and electron microscopy for evidence of exfoliated IBCs and filamentous bacteria. Evidence of IBCs was found in 14 of 80 (18%) urines from women with UTI. Filamentous bacteria were found in 33 of 80 (41%) urines from women with UTI. None of the 20 urines from the asymptomatic comparative group showed evidence of IBCs or filaments. Filamentous bacteria were present in all 14 of the urines with IBCs compared to 19 (29%) of 66 samples with no evidence of IBCs (*p* < 0.001). Of 65 urines from patients with E. coli infections, 14 (22%) had evidence of IBCs and 29 (45%) had filamentous bacteria, while none of the gram-positive infections had IBCs or filamentous bacteria.

**Conclusions:**

The presence of exfoliated IBCs and filamentous bacteria in the urines of women with acute cystitis suggests that the IBC pathogenic pathway characterized in the murine model may occur in humans. The findings support the occurrence of an intracellular bacterial niche in some women with cystitis that may have important implications for UTI recurrence and treatment.

## Abstract 2

Analyzing urine specimens from women with bladder infections, Scott Hultgren and colleagues find evidence for intracellular bacterial communities, which have been associated with recurrent urinary tract infections in mice.

## Editors' Summary

**Background.:**

Every year, nearly 10 million people in the United States—mainly women—consult their doctors because of a urinary tract infection (UTI). UTIs occur when bacteria living in the gut—usually Escherichia coli—get transferred to the opening of the urethra (the tube through which urine leaves the body), as may occur during sexual intercourse. From here, the bacteria can move into the bladder (the muscular sac that stores urine until it is excreted) where they can multiply and cause cystitis (inflammation of the bladder). If cystitis is untreated, the bacteria can move further up the urinary tract and infect the kidneys (which make urine). Symptoms of UTIs include pain when urinating, frequent and intense urges to urinate, and cloudy urine. UTIs are diagnosed by looking for bacteria and white blood cells (that fight infection) in the urine; the usual treatment is a short course of antibiotics.

**Why Was This Study Done?:**

Half the women who get a UTI will have another attack within a year, often caused by the same bacterial strain. It is generally thought that these strains persist in the gut and reinfect the urinary tract, but recent animal studies suggest an additional explanation. In mice, E. coli strains that cause UTIs can invade the cells lining the bladder. Here, they replicate and form so-called intracellular bacterial communities (IBCs). Many of the infected cells fall off the bladder's surface into the urine, but IBCs also release bacteria, many of which have a long, slender filamentous appearance (E. coli usually have a simple rod-like shape). Immune system cells normally kill bacteria in the urine but cannot deal with filamentous bacteria. In mice, these bacteria can then reinfect the lining of the bladder and establish long-lasting intracellular reservoirs of bacteria that are protected from antibiotics and probably from the host immune system. If this IBC cycle occurs in people, it might explain why some UTIs recur and might suggest ways to manage these recurrences. In this study, therefore, the researchers have investigated whether there is an IBC cycle in women.

**What Did the Researchers Do and Find?:**

The researchers collected urine from 80 young women with cystitis and from 20 women with no symptoms who had had cystitis previously. They identified the type of bacteria in each sample and looked for IBCs and filamentous bacteria using light microscopy, electron microscopy, and a technique called immunofluorescence. None of the women without cystitis had IBCs or filamentous bacteria in their urine, but IBCs were found in nearly 1 in 5, and filamentous bacteria were in nearly half, of urine samples from the women with cystitis. All the urine samples that contained IBCs also contained filamentous bacteria. All of the women with IBCs and most of them with filamentous bacteria had *E coli* infections. Finally, the women with IBCs and filamentous bacteria in their urine had higher bacterial counts in their urine and had symptoms of cystitis for slightly longer than those without.

**What Do These Findings Mean?:**

These findings suggest that the IBC cycle identified in mice occurs in at least some women with UTIs and may be associated with infections caused by E. coli. Because only one urine sample was collected from each woman, the cycle may be more common than these findings suggest. That is, in some cases the sample may have been taken at a time when there were no IBCs or filamentous bacteria in the urine. Also, because samples were taken at only one point in time, this study does not show whether intracellular bacteria persist and contribute to recurrent UTIs in women, as they appear to do in mice. To provide more information about the IBC cycle in people and its clinical relevance, additional studies are needed to examine whether there are any associations between the presence of IBCs and filamentous bacteria and treatment responses and recurrence, and to examine what is actually happening in the bladder during UTI. Until such studies are done, the clinical implications of the current findings remain uncertain. However, one possibility is that the presence of IBCs and filamentous bacteria in urine might identify people who would benefit from longer treatment with antibiotics or treatment with antibiotics that kill bacteria inside human cells.

**Additional Information.:**

Please access these Web sites via the online version of this summary at http://dx.doi.org/10.1371/journal.pmed.0040329.

- The MedlinePlus encyclopedia contains pages on urinary tract infection, on cystitis, and on recurrent cystitis (in English and Spanish)
- Information is available from the UK National Health Service Direct health encyclopedia on urinary tract infections and on cystitis
- The US National Kidney and Urologic Diseases Information Clearinghouse provides information on urinary tract infections (in English and Spanish)
- Information is available from the American Urological Association on urinary tract infections in adults

## Introduction

Urinary tract infections (UTIs) affect nearly 13 million women annually in the United States alone and can result in significant costs and morbidity [1–5]. Uropathogenic Escherichia coli (UPEC) is the predominant causative agent, responsible for up to 85% of community-acquired infections [6,7]. The majority of UTIs are thought to arise when uropathogens present in the fecal flora colonize the vaginal introitus, ascend into the bladder, and initiate a host response manifested by secretion of cytokines, pyuria, and the onset of symptoms [8].

Women have a 25% chance of experiencing a recurrent UTI within six months [9] of an index episode and a 44% chance of recurrence within one year [10] despite appropriate therapy of the initial infection and negative follow-up urine cultures. Over one-half of all recurrent episodes of acute uncomplicated cystitis are caused by the same bacterial strain as the initial infection [11,12]. As with initial UTIs, it is widely thought that recurrences occur through reascension and reinoculation of the bladder lumen by a UPEC strain that has persisted in the periurethral or fecal flora following the previous UTI.

**Figure 1:**
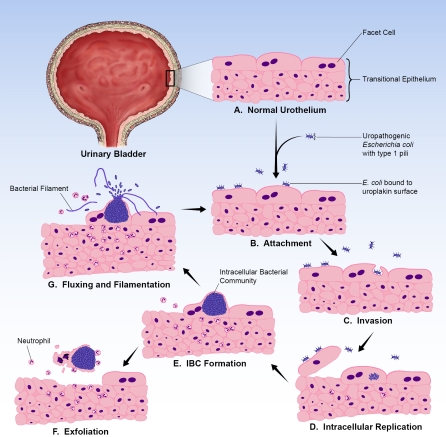
UPEC IBC Pathogenic Pathway Observed in the Murine Cystitis Model The bladder urothelium (A) is a pseudostratified transitional epithelium lined by large facet cells. These cells have an apical asymmetric unit membrane containing uroplakins that help form the impermeable bladder barrier and also serve as receptors for UPEC. Bacteria introduced into the bladder adhere to the bladder surface via type 1 pili (B). Upon attachment, bacteria are able to invade (C) and replicate (D) within the facet cell cytoplasm. UPEC form large biofilm-like IBCs within these cells (E). Ultimately the bacteria flux out of their intracellular niche (G), some adopting a filamentous morphology; they then adhere to other host cells and re-enter the infectious cycle. During this process, infected urothelial cells are sloughed into the urine (F) and neutrophils are recruited to the site of infection.

Recently, it was demonstrated in a murine model of cystitis that UPEC utilize a multistep pathogenic cycle during infection in which they progress through an intracellular niche within the bladder (Figure 1) [13–15]. UPEC express adhesive fibers known as type 1 pili that mediate binding to and invasion of luminal facet cells of the bladder [16–18]. This intracellular niche is conducive to UPEC replication and formation of intracellular bacterial communities (IBCs) with biofilm-like properties [13]. IBCs exist only transiently before the bacteria dissociate and migrate out of the facet cell, many adopting a filamentous morphology [14]. The filamentous UPEC avoid engulfment by neutrophils, thus allowing them to reinvade the urothelium [14]. Upon infection, the host exfoliates and expels bladder epithelial cells into the urine. Ultimately, UPEC are able to form quiescent intracellular reservoirs composed of small rosettes of bacteria within Lamp-1–positive endocytic vesicles that can persist for several weeks protected from antibiotics and, presumably, undetected by the host immune system [15,19,20]. Epithelial turnover may cause the quiescent bacteria to revert to an actively replicative form, leading to recurrent bacteriuria [19].

To date, an intracellular bacterial pathway has not been investigated in humans. In this study, with the knowledge that superficial bladder cells exfoliate in response to infection, we analyzed urine samples from women with acute, uncomplicated cystitis and from asymptomatic women to investigate whether the IBC and/or filamentous ex vivo hallmarks of the IBC cycle were present.

## Methods

### Specimen Collection

Specimens were collected from women between the ages of 18 and 49 y who were enrolled in various studies of acute cystitis at the Student Health Center of the University of Washington in Seattle, Washington between January 2005 and January 2007. Urine samples were collected from 80 women with acute cystitis prior to treatment and a comparative group of 20 different asymptomatic women seen for follow-up at least 1 wk after successful cystitis treatment. Exclusion criteria for both groups included antibiotic use in the prior week, phenazopyridine use in the prior 2 d, symptoms or signs of pyelonephritis, pregnancy, chronic illness such as diabetes, recent catheterization, or known anatomic or functional abnormalities of the urinary tract. Subjects provided midstream, clean-catch urine specimens for culture and analysis and questionnaire data that included information about their general health, current UTI episode, and UTI and sexual histories. All studies were approved by the Human Subjects Review Committee at the University of Washington and all participants provided written informed consent. A small number of urine samples from women with acute cystitis at the Medical Campus Student Health Clinic at Washington University in St. Louis, Missouri were also collected for optimization of microscopy and staining methods. The collection and analysis of all specimens was approved by the Washington University Human Research Protection Office.

Urines were cultured by standard methods and acute cystitis isolates were banked at the University of Washington. A woman was considered to have a UTI if she had urinary symptoms and a urine culture of ≥10^2^ CFU/ml of a uropathogen [21]. Asymptomatic bacteriuria was defined as the presence of ≥10^5^ CFU/ml of a uropathogen in a woman with no urinary symptoms. Urine hemocytometer white blood cell (WBC) counts were determined using Kova Glasstic Slide 10s with Grids (Hycor Biomedical). Within 1 h of micturition, urines were separated into three aliquots that were fixed with 1% final concentration of formalin or 2.5% final concentration of glutaraldehyde, or were left unfixed and sent overnight on ice to Washington University in St. Louis, Missouri. To reduce potential bias, no clinical information was sent to investigators at Washington University prior to examination of the urine specimens for IBCs and filamentous bacteria.

### Light Microscopy

Fixed and unfixed urine samples were cytocentrifuged for 6 min at 1,000 rpm onto poly-L-lysine-coated glass slides using a CytoPro 7620 cytocentrifuge (Wescor). Samples were then briefly heat fixed and stained with filter sterilized Protocol Hema 3 stains (Wright-Giemsa method, Fisher Scientific). Between 800 μl and 5 ml of each sample were screened by light microscopy using an Olympus BX51 light microscope (Olympus America).

### Immunofluorescence

Slides of unfixed and formalin-fixed urine specimens were prepared as described above, washed in filter-sterilized PBS, blocked in 1% BSA/0.3% Triton X-100 for 1 h at room temperature, and subsequently incubated for 1 h with rabbit anti-E. coli (1:1,000, cross reacts with other *Enterobacteriaceae*, US Biological) and goat anti-uroplakin III (1:100, Santa Cruz Biotechnology) primary antibodies. After three washes in PBS for 5 min and staining with Alexa Fluor 488-, 594-, or 546-conjugated donkey secondary antibodies (1:1,000, Molecular Probes) for 30 min, slides were washed, stained with Hoescht or TOPRO-3, coverslipped with Fluoromount G (Southern Biotechnology), and examined using an epifluorescent Zeiss Axioskop or Zeiss LSM410 confocal laser scanning microscope (Carl Zeiss).

### Electron Microscopy

Electron microscopy was utilized to gain higher resolution images of bacteria and IBCs in human urines. For transmission electron microscopy (TEM) and immunostaining, glutaraldehyde-fixed urine specimens were gently pelleted and embedded in 1% agarose. Samples were processed and immunostained as previously described using rabbit anti-E. coli antibody (1:200) followed by 18 nm colloidal gold-conjugated anti-rabbit IgG (1:30, Jackson ImmunoResearch Laboratories) [16]. All labeling experiments were conducted in parallel with controls in which primary antibodies were omitted. Sections were viewed on a JEOL 1200 EX transmission electron microscope (JEOL USA) at 80 kV accelerating voltage.

For scanning electron microscopy (SEM), glutaraldehyde-fixed urine specimens were cytocentrifuged as described above onto 1% polyethylenimine-coated, 12 mm, round, glass coverslips. Processing of samples and SEM were done as previously described [16] and viewed with a Hitachi S-450 scanning electron microscope (Hitachi) at 20 kV accelerating voltage.

### IBC and Filament Criteria

Urine samples were screened for the presence of IBCs and filamentous bacteria by light microscopy and, if positive, verified by immunofluorescence. Specimens were considered positive for IBCs if large, dark-staining epithelial cells were observed containing collections of what appeared to be intracellular bacteria. Positive staining with anti-uroplakin antibodies detected by immunofluorescence and fluorescent intensity profiles were used as confirmation that bacteria were located within facet cells. Filamentation was confirmed using immunofluorescent staining and measurements were made to determine whether bacteria over 20 μm in length were present.

### Mouse Infection Studies

An E. coli isolate from a cystitis patient with findings of urine IBCs and filamentous bacteria was inoculated by transurethral catheterization into the bladders of 8-wk-old female C3H/HeN mice (Harlan-Sprague Dawley) as previously described [17] in order to compare human and mouse urine cytology. Mouse urine samples were collected 30 h postinoculation by bladder massage over a sterile 1.5 ml Eppendorf tube, cytocentrifuged, and stained as described above. Mice were humanely killed and bladders were immediately aseptically removed, fixed in neutral buffered formalin, paraffin embedded and sections were stained with hematoxylin and eosin (H&E).

### Statistical Analysis

Categorical variables were compared using Pearson Chi-square and Fisher exact tests as appropriate. Continuous variables were compared using the Mann-Whitney U test since these variables were not normally distributed. All tests were two-tailed and a *p*-value less than 0.05 was considered significant. Analyses were performed using SPSS (version 14.0) and SAS (version 9.0).

## Results

### Study Population

The urines of 100 women at the University of Washington were analyzed in this study, 80 with acute uncomplicated cystitis and 20 asymptomatic women with a history of recent UTI. The median age of these 100 women was 22 (range 18–41) and the majority identified themselves as white (75%) and never married (77%). This population reported a median of 3 (range 0–30) previous UTIs, and 53% reported having had sexual intercourse in the 24–48 hours prior to sample collection.

### Urine Cultures

The pathogens cultured from the 80 urines obtained from women with UTI included 65 (81%) E. coli, four (5%) Staphylococcus saprophyticus, three (4%) *Enterococcus*, two (3%) each of Enterobacter aerogenes, Klebsiella pneumoniae, and Proteus mirabilis, and one (1%) *Citrobacter diversus.* There was also one (1%) UTI with high levels of both E. coli and S. saprophyticus. None of the 20 comparative asymptomatic women had significant bacteriuria.

### Light Microscopy, Immunofluorescence, and Electron Microscopy

**Figure 2:**
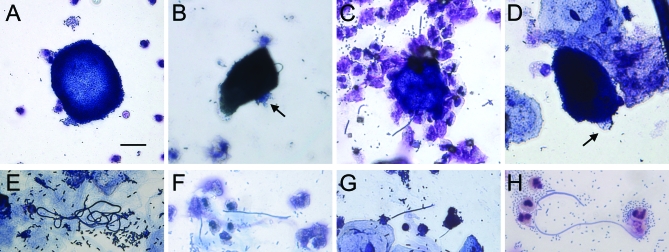
Light Microscopy Findings of Potential IBCs and Filaments in Urines from Women with Cystitis Light microscopy screening of urines from cystitis patients revealed biofilm-like collections of bacteria (A), potential intracellular bacterial communities (B–D) and filamentous bacteria (E–H). Many large biofilm-like collections of small, morphologically coccoid bacteria were found in cystitis urine samples. Dark-staining epithelial cells with potential IBCs were observed often with bacteria that appear to be protruding from within (arrows). Tangled collections and individual long filamentous bacteria were also found in several of the samples. Scale bar, 20 μm, applies to all photomicrographs.

Light microscopic analysis of some urine samples revealed large biofilm-like collections of morphologically coccoid bacteria, often in association with cell nuclei or debris (Figure 2A). Analysis also revealed large, dark-staining, often binucleate cells containing what appeared to be intracellular bacteria in several samples (Figure 2B–2D). Bladder facet cells are typically binucleate and appear large. Bacteria were sometimes observed seemingly protruding or exiting from these cells. Additionally, long filamentous bacteria were seen in many of the urine samples (Figure 2E–2H). Neutrophils, epithelial cells, and morphologically normal bacteria were also observed in the majority of specimens.

**Figure 3:**
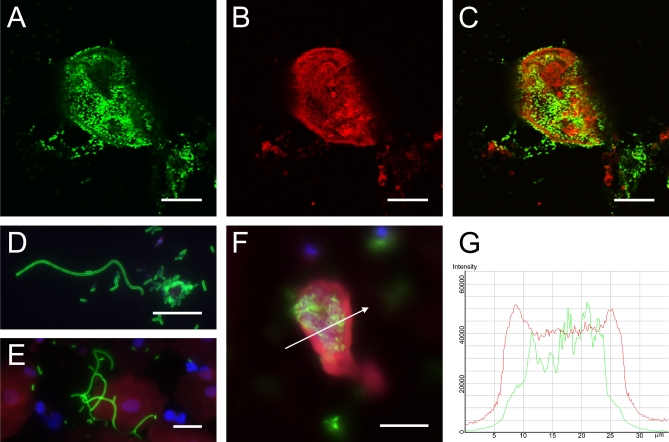
Immunofluorescence Confirmation of IBCs and Filaments in Urines from Women with Cystitis Urines from women with cystitis were stained with antibodies against E. coli (green) and uroplakin III (red). Confocal microscopy analysis revealed large collections of bacteria (A) and cells with partial membrane and cytoplasmic uroplakin staining (B). Merged images (C) show these bacteria to be intracellular. Filaments of the uropathogen over 20 μm in length were also observed in many of the urine samples (D and E). To quantify fluorescence, a slice was taken through the middle of an IBC (F) and fluorescent intensity was analyzed along a traversing line (arrow). A representative fluorescent intensity distribution profile (G) shows peaks of uroplakin (red) staining corresponding to the facet membrane and E. coli (green) staining localized intracellularly. Scale bars, 20 μm.

Immunofluorescence verified the presence of urine IBCs and filaments (Figure 3). Cells were stained with antibodies to E. coli (Figure 3A) and uroplakin III, a marker that stains facet cell membrane and cytoplasmic vesicles (Figure 3B). Merged images (Figure 3C) revealed communities of bacteria within exfoliated urine facet cells. The distribution of fluorescent staining was defined quantitatively by generating profiles of fluorescent intensity along lines traversing the middle of IBCs (Figure 3F and 3G). IBCs generally had higher peaks of uroplakin staining (red) for the cellular membrane while the uropathogen staining (green) was primarily localized within the cell itself between the membrane peaks. In addition, filamentous bacteria were stained (Figure 3D and 3E) and measured to verify multiple bacterial filaments longer than 20 μm in a given positive sample.

**Figure 4:**
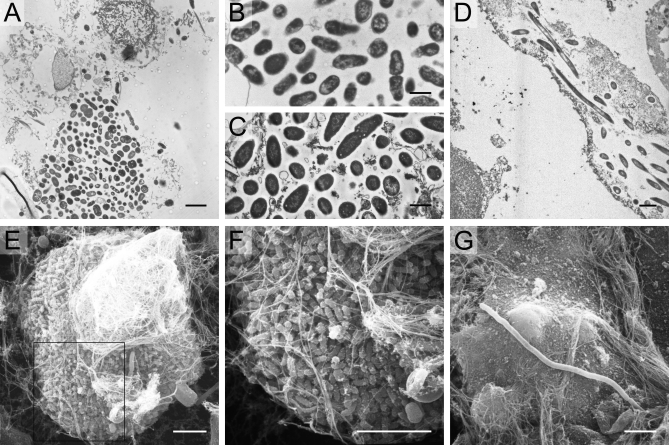
Electron Microscopy Findings in Urines from Women with Cystitis TEM analysis of human cystitis urine specimens (A) revealed large collections of bacteria associated with nuclei and other cellular debris. These collections of bacteria from human urines (B) have similar morphology and organization as those recovered from intact murine intracellular bacterial communities (C). Bacteria and filaments were also observed intracellularly within exfoliated epithelial cells in a urine sample quickly fixed and analyzed from an E. coli cystitis patient (D). SEM analysis of cystitis urines deemed positive for IBCs and filaments captured large bacterial biofilm-like collections (E and F) composed of bacteria with a smaller, more coccoid morphology than typical E. coli. Long filaments were also captured by SEM (G). Scale bars, 2 μm (A and D), 1 μm (B and C), and 5 μm (E–G).

SEM and TEM were used to generate higher-resolution images of IBCs and filaments (Figure 4). Large collections of bacteria were often observed associated with nuclei, lipid membrane, or other cellular debris (Figure 4A). The size, morphology, and spacing of the bacteria in these large collections in human urines (Figure 4B) were similar to what has been observed in murine urine samples (Figure 4C). Intracellular bacterial filaments were also found within an exfoliated epithelial cell from an E. coli urine specimen (Figure 4D). By SEM, large spherical biofilm-like collections of bacteria (Figure 4E and 4F) were observed in fixed urine positive for IBCs and filaments. Higher magnification revealed that these bacteria adopted a smaller, more coccoid morphology as typically seen in mouse IBCs. Long filamentous bacteria were also found in these samples (Figure 4G). Immunoelectron microscopy of these E. coli urine specimens with anti-E. coli antibody demonstrated positive staining of the large collections of bacteria and filaments.

### Comparison of Cystitis and Asymptomatic Subjects

Women with cystitis had higher urine WBC counts, more frequently reported recent sexual intercourse, and were slightly older than comparative subjects. The two groups were similar in race, education, marital status, and number of previous UTIs. IBCs were detected in 14 (18%) and filamentous bacteria in 33 (41%) of the 80 urine specimens from women with acute cystitis. None of the 20 urines from the comparative group showed evidence of IBCs or filaments (Table 1).

**Table 1:**
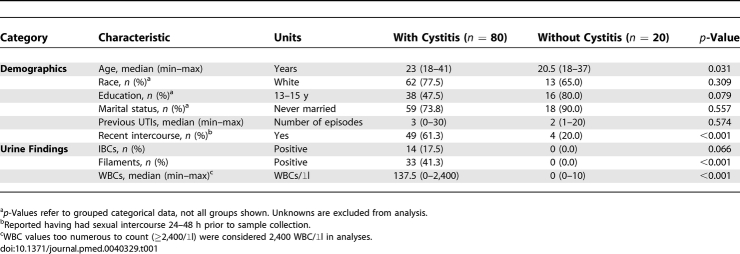
Characteristics of Women with Acute Cystitis versus Asymptomatic Women

### Characteristics of Urines from Women with Cystitis

All 14 (100%) of the urines with IBCs had filamentous bacteria compared with 19 of 66 (29%) urines without IBCs (*p* < 0.001). IBCs and filaments were observed only in urines with gram-negative uropathogens, with none of the gram-positive urines showing such evidence (*p* = 0.038). Every specimen that contained IBCs and the majority of specimens positive for filamentous bacteria (30 of 33, 88%) were from UTIs caused by E. coli. Filamentous bacteria were also observed in urine samples from women with cystitis caused by E. aerogenes, K. pneumoniae, and P. mirabilis.

### Comparison of Cystitis Patients with and without Urine IBCs and Filaments

To determine whether evidence of IBCs and filaments was associated with any patient or UTI characteristics, cystitis patients with and without IBCs or filaments were compared. IBCs and filaments were associated with higher bacterial burdens (*p* = 0.014) and a longer duration of symptoms (*p* = 0.007) (Table 2). In addition, patients with urine IBCs or filaments were slightly but significantly younger than those without these findings (*p* = 0.032) (Table 2). There were no statistically significant differences in other demographic or behavioral factors (Table 2).

**Table 2:**
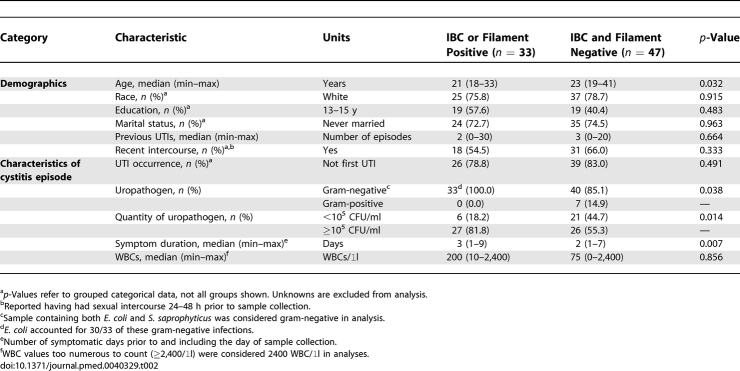
Comparisons of Cystitis Subjects With and Without Urine IBCs or Filaments

### Comparison of Urine Cytology in Human and Murine UTIs

**Figure 5:**
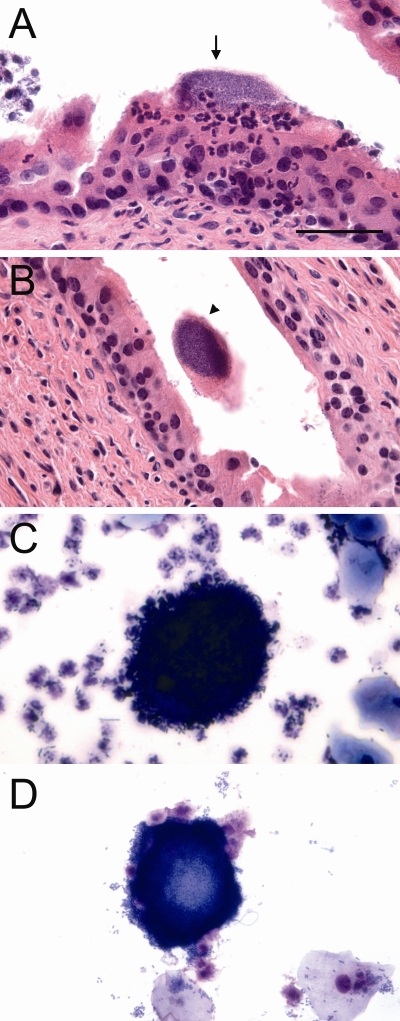
Mouse Trial and Comparison of Human and Mouse Urine The E. coli isolate from a urine specimen positive for IBCs and filaments was inoculated into mice where it progressed through the IBC pathogenic cycle. Several IBCs were observed by H&E in the mouse bladder at 30 h postinfection (A, arrow). IBCs could also be seen exfoliated into the bladder lumen (B, arrowhead). Urine collected from mice at this time point were positive for IBCs (C). These IBCs were similar in morphology and size to those formed by the same E. coli isolate in the original human urine specimen (D). Scale bar, 50 μm, applies to all photomicrographs.

Histologic examination of mouse bladders infected with an E. coli strain isolated from a woman with acute cystitis revealed IBCs in superficial facet cells present in the tissue (Figure 5A), and sloughed into the lumen (Figure 5B). Filamentous bacteria and a robust inflammatory response were also observed. The urines of these mice contained several large, dark-staining IBCs (Figure 5C) that appeared morphologically indistinguishable from those of the original human urine specimen (Figure 5D).

## Discussion

Our study provides evidence that episodes of E. coli cystitis in humans may involve an IBC pathogenic pathway similar to that observed in mice. Evidence of filamentous bacteria and exfoliated bladder facet cells containing large collections of E. coli were observed by light microscopy and confirmed by immunofluorescence. High-resolution electron microscopy showed large biofilm-like IBCs and long filamentous bacteria. The expected variety of uropathogens [6] was cultured from infected women in the study, yet IBCs and filaments were not observed in urines from women infected with gram-positive pathogens or in urines from asymptomatic women. Additionally, the urine cytologies observed in human and murine UTIs were indistinguishable.

Previous studies support our findings of an intracellular bacterial niche during UTI. In one study, human bladder biopsies from 33 women with recurrent urinary tract symptoms were analyzed after antibiotic therapy [22]. Sixteen of these patients had sterile urine cultures; however, bacteria were cultured from 8 patients' biopsies. These findings suggest that urine culture results may not necessarily reflect the true bacteriologic status of the bladder epithelium and that there may be a persistent niche for uropathogens associated with bladder tissue. In addition, multiple studies have shown that UPEC strains are able to invade and replicate within human urothelial cell lines [15,16,23]. More recently, it was demonstrated that the majority of UPEC isolates from patients with various clinical syndromes of UTI are competent for IBC formation in the murine cystitis model [24].

The ability to form filaments is an important virulence property that facilitates persistence in the murine cystitis model [25]. Filamentation can be the result of the gram-negative bacterial SOS response, which is induced by a variety of stressful stimuli, including antibiotics [26]. In this study, however, patients had no recent history of antibiotic usage. Also, filamentation did not correlate with WBC counts in the urines of these patients, as one might expect if this were a nonspecific stress response. Thus, the filaments likely represented bacteria that had emerged from an intracellular niche as seen in the murine model. The filamentation event has been shown to be triggered by TLR-4-dependent inflammatory responses in the murine model of cystitis [25], however, whether this is also true in the human remains to be determined. We were able to capture electron microscope images of these filamentous bacteria within the intracellular niche of sloughed urothelial cells. In addition, the urine finding of filamentous bacteria significantly correlated with the presence of IBCs in these samples. This correlation could be explained by the association of these two entities in the same pathogenic pathway or may be due to the higher bacterial burden in these samples and an enhanced ability to detect these endpoints by microscopy.

Interestingly, the presence of IBCs or filaments was associated with patients who had significantly longer self-reported durations of symptoms. This finding may relate to the kinetics of the IBC cycle and the time point at which IBCs are exfoliated into the urine, or it may be related to the higher burden of bacteria in the urines at these time points. Each urine analyzed represents a single point in what may be a temporally regulated pathogenic pathway. IBCs and filaments are likely transient [14] and, thus, could be missed if the sampling interval is not appropriate. In addition, the volume of urine analyzed represents a small proportion of the total sample micturated. Thus, the findings in this study may underestimate the prevalence of the IBC pathway.

Exfoliated cells found in the urine, while a useful reflection of the bladder tissue, have generally lost considerable structural integrity. Bacterial invasion into these damaged cells after they have been shed from the urothelium, while possible, seems unlikely. Mistaking such rare events for IBCs is implausible because samples were fixed upon micturition and bacteria would have needed significant time to multiply into large biofilm-like communities. While bladder biopsies are usually contraindicated in actively infected patients, future studies could assess biopsies from selected women with a history of recurrent UTI for the presence of an intracellular reservoir. As is observed in the murine model [15,19,20], a quiescent intracellular bacterial reservoir forms within the transitional epithelium. In this model, epithelial turnover and differentiation induce the bacteria within this reservoir to emerge and initiate the formation of new IBCs and recurrent bacteriuria. Reservoir formation, not explored in this study, could possibly serve as a seed for recurrence in same-strain UTIs in some women.

This study involved a large number of well-characterized young healthy women with acute cystitis and provides strong evidence that IBCs and filamentous bacteria can be found in this group of women. However, there are also several limitations to this study. We cannot extrapolate our results to women with different demographic characteristics or with different clinical syndromes, such as asymptomatic bacteriuria, pyelonephritis, or catheter-associated infections. We were not able, because of treatment considerations, to collect serial specimens from these women with acute cystitis, and thus we could not optimize collection time points when IBCs and filaments might be most abundant. The patients were not followed prospectively and thus we cannot evaluate important temporal associations between presence of IBCs or filaments and response to treatment and patterns of recurrence. It is possible that the sensitivity of our assays might have been better if we had been able to analyze urines quickly without the agitation of shipping. The low number of non-E. coli infections makes it difficult to assess the ability of other uropathogens to form filaments and IBCs during human infection. Lastly, it was outside the scope of this study to perform genetic analyses of bacteria collected, as has been done in previous studies [24,27]. Thus, potential genetic differences and other characteristics of bacteria collected in this study are not known at this time.

Despite these limitations, our data provide compelling evidence that there is an association between IBCs, filaments, and acute uncomplicated cystitis in young women and are suggestive of an IBC pathway in a subset of these women. In this pathway in mice, bacteria are able to invade and replicate within the urothelium where they are largely protected from host innate immunity, which may explain how the relatively few bacteria introduced into the bladder with urethral milking or sexual intercourse [28–30] are able to survive and multiply to numbers high enough to elicit symptoms in the host. The implications of these observations with regard to clinical management remain unclear. Urine IBCs and filaments may be prognostic indicators of specific clinical outcomes, or women with these findings may benefit from different management or prevention strategies such as longer treatment or use of antimicrobials with better intracellular penetration. It is imperative that additional studies with appropriate patient follow-up be conducted to address these specific questions. Further understanding of the IBC pathogenic pathway in human infection may provide new potential targets and approaches to the treatment and prevention of UTI.

## Abbreviations

IBC: intracellular bacterial community
SEM: scanning electron microscopy
TEM: transmission electron microscopy
UPEC: uropathogenic *Escherichia coli*
UTI: urinary tract infection
WBC: white blood cell
